# Comparison of Treatment Outcomes of Cavitary Mycobacterium avium Complex Pulmonary Disease with Streptomycin or Amikacin Use

**DOI:** 10.1128/spectrum.04741-22

**Published:** 2023-04-06

**Authors:** Seong Min Kim, Yong Pil Chong, Hyun Joo Lee, Tae Sun Shim, Kyung-Wook Jo

**Affiliations:** a Division of Pulmonology and Critical Care Medicine, Department of Internal Medicine, University of Ulsan College of Medicine, Asan Medical Center, Seoul, Republic of Korea; b Department of Infectious Diseases, Asan Medical Center, University of Ulsan College of Medicine, Seoul, Republic of Korea; c Department of Radiology, Asan Medical Center, University of Ulsan College of Medicine, Seoul, Republic of Korea; AP-HP

**Keywords:** *Mycobacterium avium* complex, cavitary nodular bronchiectatic, fibrocavitary, culture conversion, streptomycin, amikacin

## Abstract

The comparative outcomes of specific aminoglycosides in cavitary type (fibrocavitary or cavitary nodular bronchiectatic type) Mycobacterium avium complex (MAC) pulmonary disease (PD) are unelucidated. We investigated the treatment outcomes with streptomycin or amikacin inclusion in the treatment regimen. From 2006 to 2020, 168 patients with cavitary MAC-PD who received guideline-based therapy (a three-drug oral antibiotic regimen with macrolide, ethambutol, and rifampin with an injectable aminoglycoside) for ≥1 year at a tertiary referral center in South Korea were retrospectively enrolled. We compared the rates of the culture conversion achievement of patients with streptomycin or amikacin use. Of the 168 participants, 127 patients (75.6%) received streptomycin and 41 (24.4%) received amikacin (median [interquartile range] treatment duration of 17.6 [14.2 to 25.2] and 17.0 [14.0 to 19.4] weeks, respectively). The overall culture conversion rate at treatment completion was 75.6% (127/168), and the rates were similar for the streptomycin-treated and amikacin-treated groups (74.8% [95/127] and 78.0% [32/41], respectively; *P* = 0.674). A multivariate analysis revealed that the achievement of culture conversion did not differ significantly with streptomycin or amikacin use (adjusted odds ratio, 1.086; 95% confidence interval, 0.425 to 2.777). The rate of adverse events was similar in the two groups. In conclusion, in cavitary MAC-PD, treatment with streptomycin-containing and amikacin-containing regimens results in similar rates of culture conversion achievement.

**IMPORTANCE** We found that among the participants with cavitary MAC-PD who received guideline-based treatment for ≥1 year, the selection of either streptomycin or amikacin in the treatment regimen led to similar rates of culture conversion at treatment completion. In addition, the adverse reaction development rate did not differ significantly for streptomycin and amikacin. These findings suggest that either streptomycin or amikacin can be selected for the treatment of MAC-PD, according to the physician’s or patient’s preference, such as the route of administration.

## INTRODUCTION

Guidelines for the treatment of Mycobacterium avium complex (MAC) pulmonary disease (PD) recommend different treatment modalities, depending on the presence of cavitary lesions (1, 2). The administration of three oral antibiotics (i.e., macrolide, ethambutol, and rifampin) three times per week is recommended for patients with noncavitary nodular bronchiectatic (NC-NB) type MAC-PD, whereas daily treatment with a three-drug oral antibiotic regimen and an injectable aminoglycoside is recommended for patients with cavitary disease (fibrocavitary [FC] or cavitary nodular bronchiectatic [C-NB] types).

For the selection of the injectable aminoglycoside, a clinical guideline published in 2020 recommends the nonpreferential selection of either streptomycin or amikacin (3). However, expert opinion indicates that amikacin is the preferred parenteral agent for the treatment of FC-type or severe MAC-PD, although streptomycin can be chosen as an alternative to amikacin (4). In contrast, the British Thoracic Society guideline recommends only amikacin as the injectable aminoglycoside for the treatment of MAC-PD with evidence of lung cavitation (5). Furthermore, in several expert opinions, amikacin is the only recommended injectable aminoglycoside (1, 2, 6).

The differences in the recommendation of aminoglycosides in clinical guidelines and expert opinions are possibly attributable to a paucity of research evidence from previous studies of the therapeutic effects of various aminoglycosides in MAC-PD. To date, no study has investigated the differences in the treatment response that are based on the specific injectable aminoglycoside that is used in MAC-PD (7). Therefore, in this study, we aimed to investigate the treatment outcomes of cavitary MAC-PD with streptomycin or amikacin as the injectable aminoglycoside.

## RESULTS

### Participants.

Eligibility screening identified 168 patients with cavitary MAC-PD who were treated with an aminoglycoside-containing regimen for ≥1 year, and these patients were retrospectively enrolled as participants in this study. In this study cohort, the mean age was 60.3 ± 10.1 years, 66.7% of the study participants were female, and the mean body mass index at diagnosis was 20.1 ± 2.5 kg/m^2^. Acid-fast bacillus (AFB) smear positivity was noted in 57.1% of the participants. Radiologically, 127 (75.6%) participants had C-NB type MAC-PD. A total of 127 (75.6%) patients received streptomycin, whereas the remaining 41 (24.4%) patients were treated with amikacin. With the exception of the causative organism, no statistical difference in baseline characteristics was noted between participants who were treated with streptomycin and amikacin (Table 1). The total treatment duration (median, interquartile range [IQR]) in this cohort (*n* = 168 patients) was 15.0 (13.6 to 17.0) months. The treatment duration was significantly longer in those who were treated with a streptomycin-containing regimen: 15.4 (IQR, 14.0 to 17.4) months versus 13.9 (IQR, 13.2 to 16.0) months, respectively (*P* = 0.002) (Table 1).

**TABLE 1:** Clinical characteristics, treatment duration, and treatment outcome of the 168 patients with cavitary-type Mycobacterium avium complex pulmonary disease, stratified by the injectable aminoglycoside used^*a*^

Characteristic	Total (*n* = 168)	Streptomycin (*n* = 127)	Amikacin (*n* = 41)	*P* value
Age (yrs)	60.3 ± 10.1	60.3 ± 10.9	60.5 ± 7.4	0.888
Age ≥ 65 yrs	55 (32.7%)	46 (36.2%)	9 (22.0%)	0.090
Female sex	112 (66.7%)	80 (63.0%)	32 (78.0%)	0.075
Body mass index (kg/m^2^)	20.1 ± 2.5	20.2 ± 2.5	20.0 ± 2.4	0.656
Body mass index < 18.5 kg/m^2^	40 (23.8%)	28 (22.0%)	12 (29.3%)	0.345
Current or past smoker	41 (24.4%)	35 (27.6%)	6 (14.6%)	0.094
History of tuberculosis	82 (48.8%)	67 (52.8%)	15 (36.6%)	0.072
Comorbidities				
Diabetes mellitus	11 (6.5%)	8 (6.3%)	3 (7.3%)	0.731
Chronic liver disease	12 (7.1%)	10 (7.9%)	2 (4.9%)	0.403
Chronic obstructive lung disease	10 (6.0%)	9 (7.1%)	1 (2.4%)	0.454
Malignancy	30 (17.9%)	26 (20.5%)	4 (9.8%)	0.160
Etiology				0.048
Mycobacterium avium	84 (50.0%)	69 (54.3%)	15 (36.6%)	
Mycobacterium intracellulare	84 (50.0%)	58 (45.7%)	26 (63.4%)	
Positive acid-fast bacilli smear at treatment initiation	96 (57.1%)	73 (57.5%)	23 (56.1%)	0.876
No. of involved lobes	4.0 (3.0 to 5.0)	4.0 (3.0 to 4.5)	4.0 (3.0 to 5.0)	0.061
Radiologic type				0.402
Cavitary nodular bronchiectatic type	127 (75.6%)	94 (74.0%)	33 (80.5%)	
Fibrocavitary type	41 (24.4%)	33 (26.0%)	8 (19.5%)	
Total treatment duration (mo)	15.0 (13.6 to 17.0)	15.4 (14.0 to 17.4)	13.9 (13.2 to 16.0)	0.002
Total duration of an injectable aminoglycoside usage (wks)	17.1 (14.0 to 23.5)	17.6 (14.2 to 25.2)	17.0 (14.0 to 19.4)	0.188
Culture conversion at 12 mo	123 (73.2%)	90 (70.9%)	33 (80.5%)	0.226
Culture conversion at treatment completion	127 (75.6%)	95 (74.8%)	32 (78.0%)	0.674

a Data are presented as the mean ± standard deviation, median (interquartile range), or number (%).

### Detailed treatment modality of aminoglycoside.

Of the participants, all of those who started treatment before December of 2017 were treated with streptomycin, whereas those whose treatment was initiated after December of 2017 were prescribed amikacin. The median treatment durations of the 127 patients who were treated with streptomycin and the 41 patients who were treated with amikacin were 17.6 (IQR, 14.2 to 25.2) and 17.0 (IQR, 14.0 to 19.4) weeks, respectively (*P* = 0.188) (Table 1). Streptomycin was administered intramuscularly in all cases, and intramuscular injection was the most frequent route of administration of amikacin (80.5%, 33/41).

### Adverse events of aminoglycoside.

Among the participants treated with streptomycin, 8.7% (11/127) discontinued aminoglycoside treatment due to adverse events, including hearing impairment (*n* = 3), tinnitus (*n* = 3), rash (*n* = 1), injection site pain (*n* = 1), and others (*n* = 3). Of the 41 participants who were treated with amikacin, treatment with the injectable aminoglycoside was interrupted in 6 (14.6%) participants due to new-onset adverse events, including hearing impairment (*n* = 3), a tingling sense (*n* = 1), azotemia (*n* = 1), and injection site pain (*n* = 1).

### Treatment outcome, according to the aminoglycoside used.

The overall culture conversion rate at 12 months after treatment initiation in 168 participants was 73.2% (123/168). In addition, a total of 75.6% (127/168) achieved culture conversion at treatment completion. The culture conversion rates at treatment completion were similar between streptomycin use and amikacin use: 74.8% (95/127) and 78.0% (32/41), respectively (*P* = 0.674) (Table 1).

Table 2 presents the baseline characteristics, according to the achievement of culture conversion at treatment completion. Significant intergroup differences in several baseline characteristic were noted, including sex, smoking history, history of tuberculosis, diabetes mellitus, AFB smear positivity, and radiologic type. The prescribed aminoglycoside was similar in the participants with and without culture conversion.

**TABLE 2:** Clinical characteristics and treatment duration of the 168 patients with cavitary Mycobacterium avium complex pulmonary disease, according to the achievement of culture conversion at treatment completion^*a*^

Characteristics	Total (*n* = 168)	Culture conversion at treatment completion (*n* = 127)	No culture conversion at treatment completion (*n* = 41)	*P* value
Age (yrs)	60.3 ± 10.1	60.1 ± 10.4	61.2 ± 9.4	0.554
Age ≥ 65 yrs	55 (32.7%)	40 (31.5%)	15 (36.6%)	0.546
Female sex	112 (66.7%)	94 (74.0%)	18 (43.9%)	<0.001
Body mass index (kg/m^2^)	20.1 ± 2.5	20.3 ± 2.4	19.7 ± 2.5	0.142
Body mass index < 18.5 kg/m^2^	40 (23.8%)	27 (21.3%)	13 (31.7%)	0.172
Current or past smoker	41 (24.4%)	24 (18.9%)	17 (41.5%)	0.003
History of tuberculosis	82 (48.8%)	54 (42.5%)	28 (68.3%)	0.004
Comorbidities				
Diabetes mellitus	11 (6.5%)	4 (3.1%)	7 (17.1%)	0.005
Chronic liver disease	12 (7.1%)	10 (7.9%)	2 (4.9%)	0.732
Chronic obstructive lung disease	10 (6.0%)	5 (3.9%)	5 (12.2%)	0.052
Malignancy	30 (17.9%)	20 (15.7%)	10 (24.4%)	0.209
Etiology				0.106
Mycobacterium avium	84 (50.0%)	68 (53.5%)	16 (39.0%)	
Mycobacterium intracellulare	84 (50.0%)	59 (46.5%)	25 (61.0%)	
Positive acid-fast bacilli smear at treatment initiation	96 (57.1%)	65 (51.2%)	31 (75.6%)	0.006
No. of involved lobes	4.0 (3.0 to 5.0)	4.0 (3.0 to 5.0)	4.0 (3.0 to 5.0)	0.590
Radiologic type				0.001
Cavitary nodular bronchiectatic type	127 (75.6%)	104 (81.9%)	23 (56.1%)	
Fibrocavitary type	41 (24.4%)	23 (18.1%)	18 (43.9%)	
Total treatment duration (mo)	15.0 (13.6 to 17.0)	14.9 (13.8 to 16.4)	16.5 (12.4 to 18.0)	0.698
Total duration of an injectable aminoglycoside usage (wks)	17.1 (14.0 to 23.5)	17.0 (13.6 to 21.1)	21.3 (17.1 to 29.3)	0.001
Injectable aminoglycoside				0.674
Streptomycin	127 (75.6%)	95 (74.8%)	32 (78.0%)	
Amikacin	41 (24.4%)	32 (25.2%)	9 (22.0%)	

a Data are presented as the mean ± standard deviation, median (interquartile range), or number (%).

### Univariate and multivariate analyses of treatment outcome.

As shown in Table 3, a univariate analysis showed that female sex, smoking history, history of tuberculosis, diabetes mellitus, positive AFB smear, and radiologic type were related to culture conversion at treatment completion. However, a multivariate analysis revealed that none of these factors were significant for the achievement of culture conversion. Compared with streptomycin, the use of amikacin was not associated with greater culture conversion at treatment completion (adjusted odds ratio, 1.086; 95% CI, 0.425 to 2.777; *P* = 0.863).

**TABLE 3:** Factors affecting the nonachievement of culture conversion in cavitary Mycobacterium avium complex pulmonary disease^*a*^

Factors	Univariate analysis		Multivariate analysis	
	Odds ratio (95% CI)	*P* value	Adjusted odds ratio (95% CI)	*P* value
Age ≥ 65 yrs	1.255 (0.600 to 2.624)	0.546		
Female sex	0.275 (0.132 to 0.572)	0.001	0.488 (0.149 to 1.597)	0.235
Body mass index < 18.5 kg/m^2^	1.720 (0.786 to 3.763)	0.175		
Current or past smoker	3.040 (1.416 to 6.525)	0.004	1.119 (0.334 to 3.744)	0.855
History of tuberculosis	2.912 (1.381 to 6.139)	0.005	2.078 (0.915 to 4.717)	0.080
Comorbidities				
Diabetes mellitus	6.331 (1.750 to 22.903)	0.005	4.004 (0.998 to 16.064)	0.050
Chronic liver disease	0.600 (0.126 to 2.858)	0.521		
Chronic obstructive lung disease	3.389 (0.929 to 12.362)	0.065		
Malignancy	1.726 (0.732 to 4.070)	0.213		
Etiology				
Mycobacterium avium	Reference			
Mycobacterium intracellulare	1.801 (0.878 to 3.692)	0.108		
Positive acid-fast bacilli smear at treatment initiation	2.957 (1.338 to 6.536)	0.007	2.325 (0.982 to 5.502)	0.055
Radiologic type				
Cavitary nodular bronchiectatic type	Reference		Reference	
Fibrocavitary type	3.539 (1.648 to 7.601)	0.001	1.361 (0.538 to 3.446)	0.515
Injectable aminoglycosides				
Streptomycin	Reference		Reference	
Amikacin	0.835 (0.360 to 1.936)	0.674	1.086 (0.425 to 2.777)	0.863

a CI, confidence interval.

## DISCUSSION

The incorporation of an injectable aminoglycoside is recommended in the treatment regimen for cavitary MAC-PD. However, no study has investigated whether the treatment outcome differs according to the aminoglycoside used. The treatment outcomes of FC- and C-NB-type MAC-PD were compared between participants who were treated with streptomycin and those who received amikacin in this retrospective analysis of 168 participants who underwent guideline-based therapy for ≥1 year at a tertiary referral center in South Korea. To our knowledge, this is the first study to investigate the differences in treatment outcomes based on aminoglycoside selection in MAC-PD. The key finding of the present study is that streptomycin and amikacin showed similar rates of culture conversion in the treatment of cavitary MAC-PD. The rate of adverse events was similar in the two groups that were treated with these injectable agents.

In a randomized controlled study in 2007, which was the first to demonstrate the effectiveness of an injectable drug in the treatment of MAC-PD, streptomycin was included in the treatment regimen (8). Thereafter, several studies have reported the treatment outcome of MAC-PD following treatment with an injectable aminoglycoside-containing regimen. Some studies have reported the treatment results of a streptomycin-containing regimen (9–11), and others have reported treatment responses of standard regimens that are comprised of streptomycin or amikacin (12, 13). No study has directly ascertained which of the two aminoglycosides is superior in MAC-PD. We inferred that a number of experts seem to consider the therapeutic effects of these two drugs (streptomycin or amikacin) to be similar, given that a recent guideline recommends choosing either of these drugs without preference (3). The findings of the present study appear to support this guideline.

Nevertheless, it should be noted that the therapeutic benefit of adding an injectable aminoglycoside itself could be uncertain. A randomized study in 2007 proved that a higher rate of culture conversion can be achieved by adding an injectable aminoglycoside to the three-drug oral regimen (8). In contrast, the meta-analysis conducted by Kwak et al. did not suggest the benefit of the use of an injectable aminoglycoside after finding a similar rate of treatment success, irrespective of the addition of an injectable aminoglycoside (14). Therefore, the premise needs to be emphasized that if injectable aminoglycosides do not confer any therapeutic benefit, then our result that no difference was observed between streptomycin and amikacin would be unsurprising. Although current guidelines and expert opinions recommend the incorporation of an injectable aminoglycoside to the therapeutic regimen for the treatment of cavitary type MAC-PD (1–4), the therapeutic roles of injectable aminoglycosides in MAC-PD need to be further elucidated.

The present study had notable limitations. Most importantly, this study was conducted at a single referral center and had a nonrandomized, retrospective design. Considering that a meta-analysis of 16 studies (14) showed that the treatment success rate was higher in nonrandomized controlled trials than in randomized controlled trials, it is possible that the treatment results of the present study might be exaggerated. In addition, the decision regarding treatment regimen selection and treatment duration was made by the attending physician, without adherence to a preestablished protocol. One of the findings that reflects this limitation was that the treatment duration of the injectable aminoglycosides were longer in the patients who did not achieve culture conversion (Table 2). In clinical practice, attending physicians tend to maintain an aminoglycoside treatment if patients with MAC-PD show persistent culture positivity. These patients would continue aminoglycoside treatment if culture conversion was not achieved. This treatment pattern in clinical practice appeared to be the reason for the lower rate of culture conversion, despite the prolonged use of an injectable aminoglycoside in patients with persistent culture positivity. This finding, which originated from a limitation of the retrospective study design, should not be interpreted as implying that prolonged injectable aminoglycoside treatment would not lead to a higher rate of culture conversion. Third, although it was suggested that treatment response can be predicted by the *in vitro* susceptibility testing results of amikacin, the treatment outcome according to the MIC of amikacin was not presented in the present study. Finally, regarding eligibility screening, a substantial portion of patients who started treatment were excluded, as they did not complete ≥1 year of treatment due to various reasons, such as adverse events. This is because studies assessing the outcomes of patients with MAC-PD included only those who had completed ≥1 year of treatment (11, 13, 15–17), and we thought that this enrollment criterion appeared to be the most suitable method by which to evaluate the effects of the therapeutic regimen or drug.

In conclusion, we found that among the participants with cavitary MAC-PD who received a guideline-based treatment for ≥1 year, the selection of either streptomycin or amikacin in the treatment regimen led to similar rates of culture conversion at treatment completion. In addition, the adverse reaction development rate did not differ significantly for streptomycin or amikacin. These findings suggest that either streptomycin or amikacin can be selected for the treatment of MAC-PD, according to the physician’s or patient’s preference, such as the route of administration.

## MATERIALS AND METHODS

### Participants.

**FIG 1:**
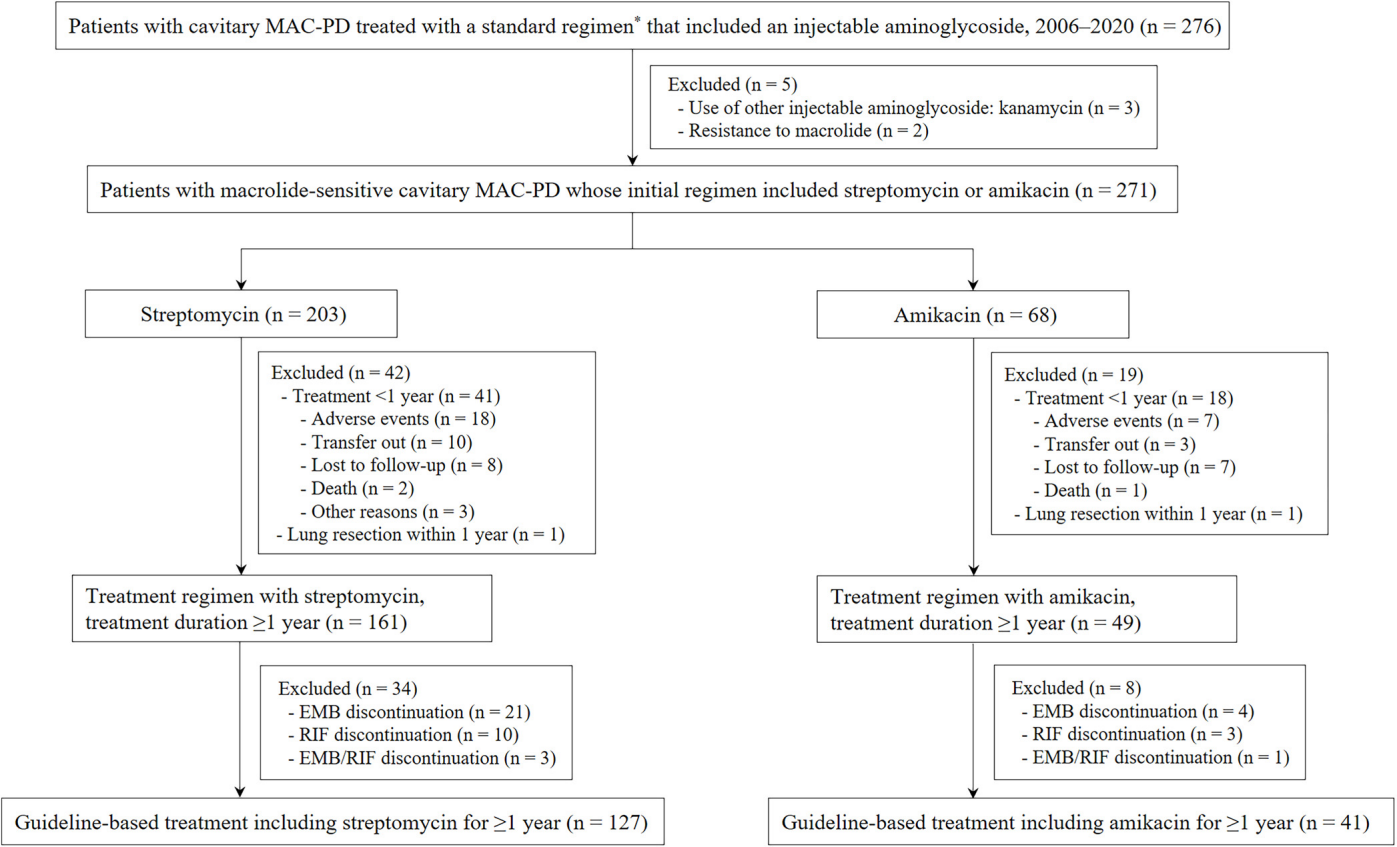
Flowchart depicting the screening and selection of patients with cavitary Mycobacterium avium complex pulmonary disease as well as participant disposition in the study. Abbreviations: MAC, Mycobacterium avium complex; PD, pulmonary disease; EMB, ethambutol; RIF, rifampin. *, the standard three-drug oral antibiotic treatment regimen included macrolide, ethambutol, and rifampin.

The participants of this study were retrospectively enrolled from the Asan Medical Center, which is a 2,700-bed referral hospital in Seoul, Republic of Korea. From 2006 to 2020, 276 patients with cavitary MAC-PD (FC and C-NB types) who had received a guideline-based treatment (defined as the use of a three-drug oral antibiotic regimen comprised of macrolide, ethambutol, rifampin, and an injectable aminoglycoside) were identified. Among them, after excluding patients (i) who received kanamycin and (ii) whose MAC isolates were macrolide resistant, 271 patients with macrolide-sensitive, cavitary MAC-PD who were treated with amikacin or streptomycin remained. Next, we selected the patients who maintained a guideline-based treatment for ≥1 year, and we excluded those (i) whose treatment duration was less than 1 year for various reasons, such as the development of adverse events, and (ii) who underwent surgical resection within 1 year after the treatment initiation. Finally, those who discontinued ethambutol and/or rifampin during the treatment course due to adverse events or intolerability were withdrawn from our analysis, as the treatment outcome of these patients could have been worse than those of the patients who completed the guideline-based treatment without regimen modification (12, 13, 18) (Fig. 1). Each participant received a macrolide throughout his or her treatment period. The medical records of the enrolled patients were retrospectively analyzed in July of 2022. The treatment outcomes were compared based on streptomycin and amikacin use.

The study protocol was approved by the Institutional Review Board (IRB) of the Asan Medical Center (IRB no.: 2022-1498), and the IRB waived the requirement for informed consent because of the retrospective study design.

### Selection, duration, and dosage of injectable aminoglycoside.

During the study period, the attending physician solely decided which aminoglycoside was chosen for the treatment of MAC-PD. Streptomycin or kanamycin was selected in all of the patients who were treated before December of 2017. However, as streptomycin has been unavailable in the Republic of Korea since December of 2017 due to stock unavailability, amikacin has been prescribed in all of the cases since 2017.

The treatment duration of the injectable aminoglycoside was determined by each attending physician, without adherence to any predetermined protocol. The aminoglycoside was administered intramuscularly or intravenously at a dosage of 15 mg per kilogram (with a maximum dosage of 1,000 mg per day). The dosage was generally 3 times per week for patients aged 60 years or older and 5 times per week (administered Monday to Friday) for those who were younger than 60 years.

### Radiological evaluation.

Based on the findings of chest computed tomography at the time of treatment initiation, the radiologic type was classified as either FC or C-NB, as previously defined (2, 11, 19). Lingula division was considered to be a separate lobe, which resulted in a total of six lung lobes for analysis (20).

### Microbiological examination.

Expectorated sputum or bronchoscopy-derived samples were cultured in both solid (Ogawa medium; Korean Institute of Tuberculosis, Republic of Korea) and liquid (Bactec 960 Mycobacterial Growth Indicator Tube; Becton, Dickinson, Sparks, MD, USA) media. AFB smears were made using Ziehl-Neelsen staining. Positive liquid cultures and colonies on solid medium were subjected to a polymerase chain reaction (PCR) assay using Seeplex Tuberculosis Detection (Seegen, Seoul, Republic of Korea) to differentiate the M. tuberculosis complex from nontuberculous mycobacteria (NTM). The NTM species were identified via the reverse-blot hybridization of the *rpoB* gene (GenoType Mycobacterium CM/AS; HAIN Life Science, Germany) (21).

### Treatment outcome analysis.

After treatment initiation, the patients were requested to submit expectorated sputum samples with at least a one-month interval until a conversion to a culture-negative specimen was documented. After the achievement of culture negativity, sputum samples were collected at intervals of two to three months until treatment completion.

Treatment outcomes were determined as sputum culture conversion, which was defined as at least three consecutive negative sputum cultures from respiratory samples that were collected at least 4 weeks apart during an antimycobacterial treatment, in accordance with the consensus statement (22). The time of conversion was defined as the date that the first negative culture was obtained. Given that previous reports considered cases of persistently positive cultures after >12 months of antibiotic treatment as treatment failures (23, 24), we first evaluated whether culture conversion was achieved at 12 months after treatment initiation (13, 15, 25). Then, the achievement of culture conversion at treatment completion was assessed (17).

### Statistical analysis.

All data are presented as the mean ± standard deviation, as the median with the IQR for continuous variables, or as a count (percentage) for categorical variables. We compared the data using an independent *t* test or a Mann-Whitney *U* test for continuous variables and a chi-square test or Fisher’s exact test for categorical variables. We selected the independent variables based on their statistical significance in a univariate logistic regression analysis. Next, we performed a multivariate logistic regression analysis via the enter method to calculate the adjusted odds ratio with a 95% confidence interval (CI). All tests of statistical significance were two-sided. *P* values of less than 0.05 were regarded as indicative of a statistically significant result. We performed all analyses using SPSS version 21.0 (IBM Corp., Armonk, NY, USA).
